# Impact of Multidrug Resistance on Tuberculosis Recurrence and Long-Term Outcome in China

**DOI:** 10.1371/journal.pone.0168865

**Published:** 2017-01-24

**Authors:** Yanni Sun, David Harley, Hassan Vally, Adrian Sleigh

**Affiliations:** 1 National Centre for Epidemiology and Population Health, Research School of Population Health, ANU College of Medicine, Biology and Environment, The Australian National University, Canberra, ACT, Australia; 2 School of Public Health & Human Biosciences, La Trobe University, Melbourne, Victoria, Australia; Chinese Academy of Medical Sciences and Peking Union Medical College, CHINA

## Abstract

Little is known about the impact of drug resistance on recurrence in TB. We conducted a cohort study to measure the impact of multi-drug resistance (MDR) on TB recurrence over nine years in Henan Province China. We reviewed medical records and conducted field interviews of 100 MDR and 150 non-MDR TB patients who were treated between 2001 and 2002. We compared long-term recurrence rates, risk factors, and outcomes in 2010 for 234 individuals who could be followed up. About one third (29.5%, 69/234) suffered recurrence after completion of treatment. The overall recurrence rate was 35/1,000 patient-years (PY), with a much higher rate (65/1,000 PY) among MDR-TB patients. MDR (HR: 2.75; CI: 1.58–4.79) and patient annual household income less than 10,000 Yuan (HR: 2.05; CI 1.11–3.80) were associated with recurrence. The mean time for recurrence among MDR-TB patients was 5.7 years, compared to 7.2 years among non-MDR-TB patients. Among the recurrence group members, 61.3% died, and 18.8% had failed treatments. We believe that the high TB recurrence rate after 9 years suggests that a high cure rate cannot accurately predict long-term outcome. We recommend that TB surveillance and control should be strengthened with a focus on MDR-TB and directly observed treatment, to reduce TB recurrence and transmission of MDR-TB.

## Introduction

Poor adherence to treatment is a well-known risk factor for tuberculosis (TB) recurrence. While there are few published data on recurrence rates, it is considered probable that drug resistance increases risk of recurrence [1–3].

Multidrug resistance (MDR), defined by resistance to both isoniazid (H) and rifampicin (R), is a major barrier to TB control [4]. Patients with MDR-TB have poor treatment outcomes compared with patients with drug-sensitive TB [5–8]. Research on the contribution of MDR-TB to TB recurrence provides evidence for targeting control efforts and more efficient use of health resources in high TB burden countries, like China.

Recurrence occurs in 3.0 to 36.0% of TB cases [1–4, 9, 10]. The average relapse rate among notified TB patients in China in 2014 was 3.0%, but the rate among MDR-TB patients was not reported [11]. China has the second highest national burden of MDR-TB (54,000) after India in 2014 [11]. Given the high burden and the low treatment cure rate for MDR-TB (48.0%), the adequacy of case management for MDR-TB requires investigation [2, 12] to aid TB control in China. The impact of MDR-TB on recurrence has not been investigated in China.

We conducted a retrospective cohort study in Henan Province, China to measure the long-term outcomes among patients who completed treatment under directly-observed treatment short-course (DOTS), with a particular focus on recurrence in MDR-TB. We reported mortality elsewhere [8]. Here we report causes of recurrence, particularly multidrug resistance, over nine years in Henan Province China.

## Methods

### Study setting

Henan Province is in the middle of China and had a population of 99.1 million people in 2010[13]. Although the incidence of TB in Henan (71.1 per 100,000 in 2010) is close to the China national average, the absolute number of notified TB cases in 2010 (68,042) was the second highest among all provinces [13]. DOTS, as recommended by the World Health Organization (WHO), was implemented progressively in Henan Province starting in 1996, and the reported coverage by county reached 100% in 2005.

### Study population and methods

Our study sample was drawn from a 2001 representative drug resistance survey (DRS) supported by the World Health Organization (WHO) in Henan Province. We chose Henan because it had the second highest number of TB and MDR-TB cases and had conducted high quality DRS in 2001. A total of 1,854 newly-registered HIV-negative, sputum smear-positive cases from 30 of 159 counties and districts, who had been treated for TB between July 2001 and June 2002, were selected at random from the DRS and resurveyed. The survey’s methods, findings for initial treatment outcomes, and 1,487 cases with culture results and complete medical records are reported elsewhere [14]. Among these 1,487 cases, there were 192 MDR-TB patients and 1,295 non-MDR-TB patients. One thousand two hundred and sixty of the 1,487 cases completed their treatment and were eligible for inclusion in the follow-up study. Among these, 112 were culture-proven MDR-TB patients and 1,148 were non-MDR-TB patients [14].

The follow-up study was powered to detect a 2-fold ratio in risk of recurrence over nine years between the MDR and the non-MDR groups, using a 2-sided significance level of 0.05 and a power of 0.8, and assuming a 40% recurrence in the MDR-TB group. The predicted recurrence rate in MDR-TB was based on literature and expert opinion of local experts at the TB Control Institute (TBCI) [9, 15–17]. Non-MDR patients were oversampled in a 2:1 ratio to increase precision. The minimum required sample sizes were 133 non-MDR patients and 67 MDR patients; the samples were increased proportionately to 150 and 100 patients, respectively, to account for loss to follow up.

**Fig 1:**
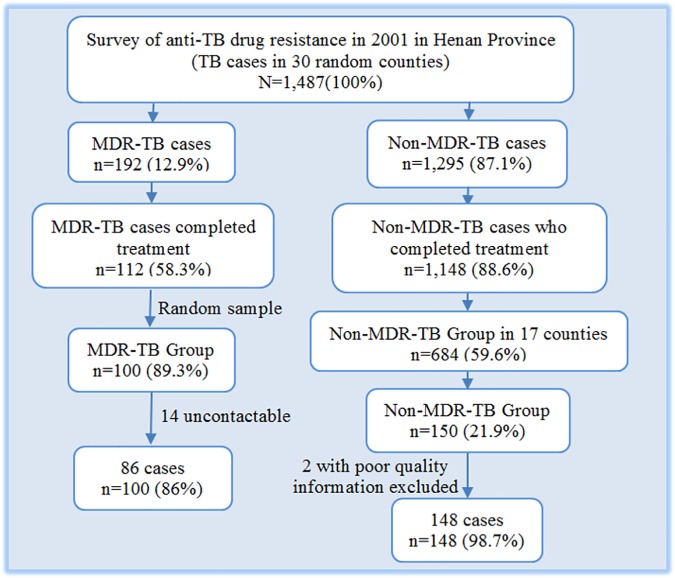
Selection of the study population in 2010 (reprinted, originally printed @BMC Public Health 2015 [8]).

Cases were selected at random from the baseline dataset. Patients surveyed in 2001 were listed, and a unique number was allocated to each individual in the dataset. A random number table was then generated using a Microsoft^™^ Excel spreadsheet. Next, a starting point in the random number table was chosen and was read for selecting cases for MDR-TB group. A corresponding number in the dataset led to a case being selected as part of the study sample. This process was repeated until 100 cases had been selected for the MDR-TB group from 17 counties and the non-MDR-TB cases were then randomly selected from the same seventeen counties by repeating the process until 150 cases had been chosen (Fig 1).

Before starting the fieldwork, a management team (2 RAs from TBCI and the first author) and an interview teams (doctors or nurses at selected county CDCs) were formed. The two RAs had been working on TB at TBCI for many years and were very familiar with local TB Control Departments (TBCDs) and staff who could facilitate the investigation. Discussion on the research content, survey methods and requirements was undertaken with them. An additional 30 skilled staff at county TBCDs were recruited as interviewers. They included 24 doctors and six nurses working at county TBCDs. All had majored in medicine, public health and nursing and had participated in one or more nationwide TB surveys.

The first author held a one-day training workshop in Zhengzhou for interviewers from study counties. The first author and the two RAs provided telephone training for those unable to attend.

Trained interviewers went to selected patients’ households on the sample list in each village. The village doctors and leaders helped interviewers to locate the interviewees in the surveyed village. The sites of interviews were usually respondents’ homes, but occasionally were in yards where they were working or at clinics. Adult family members were allowed to attend the interviews to provide supplementary information. If patients were not available for interview, other adult family members (partners, parents or siblings), or their grown-up offspring were interviewed. Each interview lasted half to one hour.

The first author and the two RAs extracted data on demographics, treatment history, drug susceptibility test (DST) results, and treatment outcomes from the DRS. All information was verified against patients’ medical records. Discrepancies found, such as age, diagnosis year and residential address, were resolved with information obtained from county or district TB dispensaries and phone calls to patients and family members. We conducted interviews from January to May 2010. Sputum status was obtained from laboratory and medical records in county or district anti-TB departments. If a patient had been reported to have died after completion of treatment in 2002, his or her medical-file information, including date and cause of death, was abstracted. If a patient could not be interviewed because they had died, moved out of the home, or were absent from the home when the interview was attempted, we interviewed family members, neighbours, village doctors, and other village leaders to collect the required information. The respondents included 129 (55.0%) patients and 105 (45.0%) family members and village doctors.

**Fig 2:**
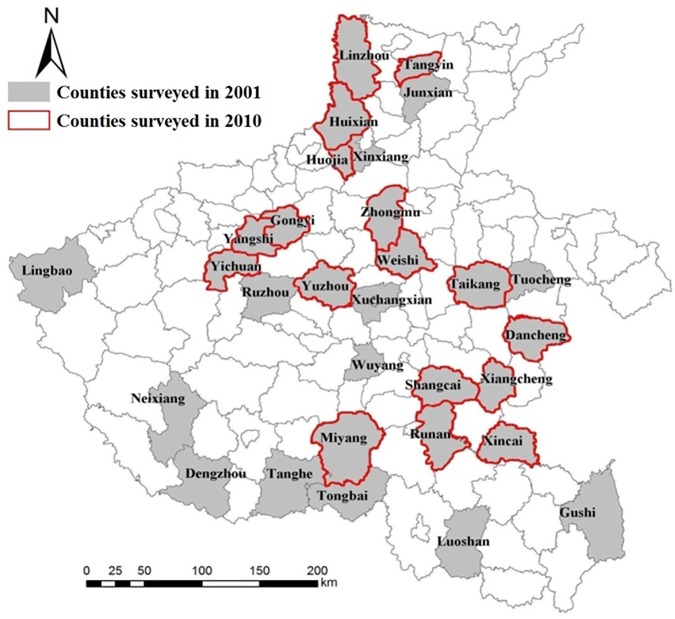
Geographical location of counties surveyed in Henan Province, 2001 & 2010.

We were unable to contact 14 of the 100 MDR-TB patients, and we excluded 2 of the 150 non-MDR-TB patients due to poor data quality. The final dataset included 86 MDR-TB and 148 non-MDR-TB patients from 17 counties (Fig 2).

### Treatment regimens and outcomes

Treatment regimens followed the Chinese National Tuberculosis Control program (NTP) treatment guidelines [18], as recommended by WHO [19]. Treatment of new smear-positive patients consisted of 2 months of H, R, pyrazinamide (Z), and ethambutol (E) followed by 4 months of H and R three times weekly. Patients who had previously received at least one month of TB treatment (i.e. retreatment patients) received 2 months of H, R, Z, S and E, followed by 6 months of H, R, and E three times weekly. Doctors directly observed drug administration at the health facility throughout treatment. The treatment regimens and outcomes have been presented in detail elsewhere [20]. Treatment outcomes were assessed using WHO definitions [19] and were recorded in routine treatment profiles. Treatment success is defined as either cure or treatment completed. Cure is defined as a patient whose sputum smear or culture is positive at the beginning of the treatment but smear- or culture-negative in the last month of treatment and on at least one occasion before this. Treatment completed need not meet the criteria for cure or failure. Failure is defined as sputum smear-positive at 5 months or later during treatment. Death is defined as a patient who dies for any reason during the course of treatment (the cause of death during TB treatment was not recorded).Recurrence was defined as a new clinical and/or microbiological diagnosis of TB in any patient who had correctly completed treatment for their first episode. Participants who had smoked before acquiring TB were considered smokers. We defined harmful alcohol consumption for males ≥ 60g of pure alcohol and females ≥ 40g of pure alcohol on average per occasion.

### Data management and statistical analysis

All data were double entered in EpiData 3.1 (The EpiData Association, Odense, Denmark, 2008), and internal consistency was determined. Internal discrepancies were checked against the original forms by the first author. Statistical inference followed standard procedures for categorical data [21]. Pearson’s χ^2^ test or Fisher’s exact test was used to test for differences in proportions. Two-sided *p*-values of <0.05 were considered significant. The overall recurrence rates, expressed as cases per 1,000 person-year (PY), were compared at two-year intervals. The follow-up period, from the date of TB treatment completion under DOTS until recurrence, death, transfer, completion of follow-up or the end of the study, was computed as the time passed since the end of TB treatment. Cox proportional hazards regression was performed with time dependant covariate in relation to TB recurrence, using a forward inclusion approach. Variables significantly correlated in the univariate model with *p*-value <0.05 and variables of epidemiological interest were included in the multivariate model. Hazard ratios (HR) and their 95% confidence intervals (CI) were used to assess association. We plotted cumulative incidence curves, unadjusted and adjusted by age and other covariates over drug resistance status. Stata software (Version 12; Statacorp) was used for data analysis and preparation of graphs.

### Ethical considerations

The original 2001 MDR-TB surveillance survey was approved by the Ethics Committee of the Department of Health of Henan Province, based on Chinese national ethical regulations. The follow-up study in 2010 was approved by the Ethics Committee of the Tuberculosis Control and Prevention Institute of Henan Provincial Center for Disease Control and Prevention and by the Human Research Ethics Committee of the Australian National University on 12 November, 2009 (Protocol 2009/553). Information sheets and consent forms were provided to participants, and consent was obtained from all study participants. Participation was voluntary, and confidentiality of data was maintained.

## Results

### Attributes of the study population

The median age of the study population was 49 years (IQR 20–84). Among the study population, 73.1% were male. A statistically significant difference in recurrence rate between MDR and non-MDR-TB patients was seen after 9 years (61.3% *versus* 27.9%; *p*<0.001). There was no significant difference in marital status between recurrent cases and others. The two groups were similar in occupation; most (≈85.0%) were farmers. Recurrent TB cases were less educated (61.1% *versus* 44.2%, *p* = 0.02), and had non-statistically-significant differences from other cases in smoking (30.7% *versus* 22.7%, *p* = 0.21) and drinking alcohol (19.4% *versus* 12.8%, *p* = 0.20). Slightly more recurrent TB cases had diabetes compared to those who did not have recurrence (*p* = 0.55). There was a trend by group of working outside for cash following TB treatment completion (5.2% *versus* 3.2%), although the difference was not statistically significant. Most patients (≈90%) paid for treatment out-of-pocket, although 88% received some financial help from community and government. Patients with recurrence were more likely to live in houses with fewer than three bedrooms (33.3% *versus* 22.2%; *p* = 0.08). Recurrence was more common in those with annual household incomes less than 10,000 Yuan (63.9% *versus* 37.4%; *p*<0.001) (Table 1).

**Table 1:** Attributes of the study population by long-term recurrence to 2010^a^.

Patient attributes		Total n = 234 (%)	Free of TB n = 165 (%)	Recurrence n = 69 (%)	*p*-value
**Median age (years) (IQR**^**b**^**)**		49 (20–84)	47 (23–84)	53.5 (20–86)	0.33
**Sex**					
	Female	63 (26.9)	48 (27.9)	15 (24.2)	
	Male	171 (73.1)	124 (72·1)	47 (75·8)	0.57
**Drug resistance status**					
Non-MDR-TB		148 (63.3)	124 (72·1)	24 (38·7)	
MDR-TB		86 (36.7)	48 (27·9)	38 (61·3)	<0.001
**Marriage status**					
	Married	185 (79.4)	137 (80.1)	48 (77.4)	
	Others ^c^	48 (20.6)	34 (19.9)	14 (20.6)	0.65
**Occupation**					
	Farmer	200 (85.8)	149 (87.1)	51 (82.3)	
	Other ^d^	33 (14.2)	22 (12.9)	11 (17.7)	0.34
**Education**					
	≥ Middle school	120 (51.3)	96 (55·8)	24 (38·7)	
	≤ Primary school	114 (48.7)	76 (44·2)	38 (61·3)	0.02
**Smoker**		58 (24.8)	39 (22.7)	19 (30.7)	0.21
**Alcohol drinker**		34 (14.5)	22 (12.8)	12 (19.4)	0.20
**Work outside** ^**e**^ **for cash after TB cured**		11 (4.7)	2 (3.2)	9 (5.2)	0.52
**Health insurance**					
	Insured	27 (11.5)	21 (12.2)	6 (9.7)	
	Own expense	207 (88.5)	151 (87.8)	56 (90.3)	0.59
**Community/government offered financial help**		195 (88.6)	143 (88.8)	52 (88.1)	0.89
**Number of bedrooms in patient’s home**					
	≥4	170 (74.9)	130 (77·8)	40 (66·7)	
	≤3	57 (25.1)	37 (22·2)	20 (33·3)	0.08
**Annual household income (Yuan)**					
	≥10,000	129 (55.6)	107 (62·6)	22 (36·1)	
	<10,000	103 (44.4)	64 (37·4)	39 (63·9)	<0.001

^a^ Those with missing values were excluded from the comparisons.

^b^ Interquartile range.

^c^ Other includes single, divorced and widowed.

^d^ Other includes worker, teacher, cadre, self-employed, housewife, students retired, no job and others.

^e^ Patient worked outside hometown for cash as a migrant worker.

### Recurrence rates

The overall recurrence rate was 35/1,000 PY (18/1,000 PY among those 10–29 years of age, and 31, 54, and 39, respectively, for those aged 30–44, 45–59, and 60 years or above). The recurrence rates were 20/1,000 PY among non-MDR-TB and 65/1,000 PY among MDR-TB cases. The highest recurrence rates were found between the eighth and ninth year after completion of treatment (74/1,000 PY), followed by the first two years (48/1,000 PY), and the third to forth (28/1,000 PY).

### Factors associated with TB recurrence

**Fig 3:**
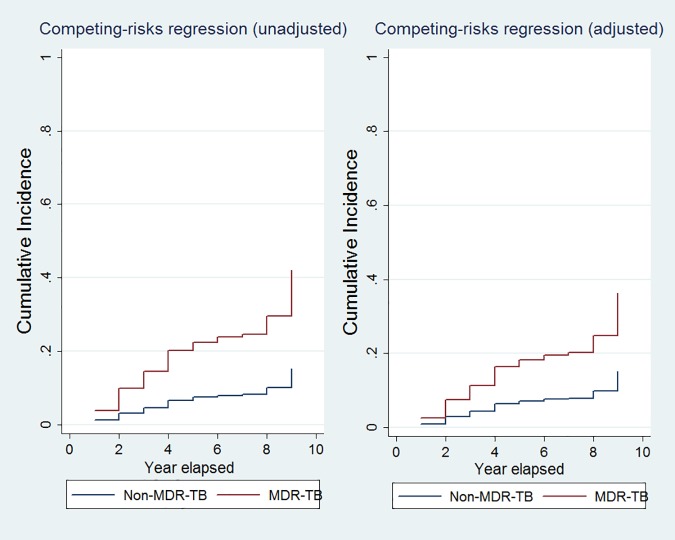
Effect of drug resistance on recurrence over time.

In univariate analysis, recurrence was significantly associated with MDR-TB, lower education attainment, having fewer than 3 bedrooms in the home, and having an annual household income less than 10,000 Yuan (Table 2). In multivariate analysis, recurrence was significantly associated with MDR (HR 2·75; 95% CI 1·58–4·79) and with an annual household income less than 10,000 Yuan (HR 2.05; 95% CI 1.11–3.80). The mean time for recurrence among MDR-TB patients was 5.7 years, compared to 7.2 years among non-MDR-TB patients (Fig 3).

**Table 2:** Factors associated with TB recurrence in the study population, 2010§.

Patient attributes		Univariate unadjusted HR (95% CI)	*p*-value	Multivariable adjusted HR (95% CI)	*p*-value
Male sex		1·04 (0·58–1·85)	0.89	0·99 (0·55–1·81)	0.98
Age groups (years)					
	≤44	1		1	
	45–59	1.48 (0.78–2.81)	0.23	1.03 (0.52–2.05)	0.93
	60~	1.27 (0.67–2.42)	0.46	0.96 (0.46–1.99)	0.92
Drug resistance status					
Non-MDR-TB		1.00		1.00	
MDR-TB		3·37 (1·98–5·73)	<0.001	2·75 (1·58–4·79)	<0.001
Education					
	≥ Middle school	1.00		1.00	
	≤ Primary school	1·95 (1·14–3·36)	0.01	1·72 (0·88–3·35)	0.11
Number of bedrooms in patient’s home					
	≥4	1.00		1.00	
	≤3	1·74 (1·01–3·00)	0.04	0·97 (0·52–1·79)	0.92
Annual household income (Yuan)					
	≥10,000	1.00		1.00	
	<10,000	2·52 (1·47–4·32)	0.001	2·05 (1·11–3·80)	0.02

§Those with missing values were excluded.

### Treatment outcomes of patients with recurrent TB on a re-treatment regimen

All patients with recurrence of TB were treated with a re-treatment regimen. Treatment outcomes are shown in **Table 3**: 40.3% (25/69) of recurrent patients were cured, 31.9% (22/69) completed treatment, 61.3% (38/69) died, 7.2% (5/69) defaulted, 18.8 (13/69) had treatment failure, and 5.8 (4/69) were lost to follow-up.

**Table 3:** Treatment outcomes of patients with recurrent TB by 2010.

Treatment outcome	Patients with recurrence n = 69 (%)
Enrolled for treatment	69 (100)
Cured	25 (40.3)
Completed	22 (31.9)
Died	38 (61.3)
Defaulted	5 (7.2)
Failed treatment	13 (18.8)
Lost to follow-up	4 (5.8)

## Discussion

In our study, about one third of participants (29.5%, 69/234) suffered recurrence after completion of treatment under DOTS. The overall recurrence rate of the followed-up patients was 35/1,000 PY, with a much higher rate (65/1,000 PY) among MDR-TB patients. We found that TB recurrence was strongly associated with MDR-TB infection and patient annual household income less than 10,000 Yuan. Moreover, MDR-TB patients had a shorter disease-free period compared to non-MDR-TB patients (5.7 vs 7.2 years, p<0.01). Importantly, the 9-year treatment outcomes at follow-up for the recurrence group were very poor, with many deaths (61.3%) and a substantial proportion of failed treatments (18.8%).

We found higher recurrence rates than in the UK (around 23/1,000 PY) [4], Malawi (3.1% of all registered TB patients between 1 July 1999 and 30 June 2000) [22], Vietnam (8.6%, 21/244 TB patients surveyed) [3], and Shanghai (3.1%, 202/6,442 over a five-year follow-up) [10]. However, the recurrence rate we found was lower than that measured in a study conducted in Uzbekistan (36.0%, 42/118 patients followed up a median of 22 months) [2]. The recurrence rate we found among MDR-TB patients was similar to that reported in Heilongjiang Province (61.0%, 79/129 patients surveyed over a four-year follow-up) [9].

We also found that MDR infection was a strong predictor of recurrence, a finding that was shown in the Uzbekistan study [2]. Long term outcomes for recurrent cases in our study were poorer than those reported in Malawi [22]. From findings reported in those studies, poor long-term outcomes may be closely associated with drug resistance status [2]. The initially-reported treatment success rate among smear-positive patients was 85.5% and was 76.6% for MDR-TB patients under DOTS in Henan [20], meeting the WHO targets of 85% and 75% [19, 23]. However, we found that about 30% of patients (61.0% of MDR-TB patients) were again diagnosed with active TB after completing treatment. Recurrence after treatment completion is common and the initially reported “cure” rate is not reliable [2].

Our results lead us to question whether the cause of recurrences of disease were true relapses or reinfection with new strains of TB. If it was the former, then DOTS did not truly cure those patients [2]. The DOTS program should be re-evaluated to determine whether drug resistance, poor adherence or low quality drugs are causes for relapse [2].

We believe that our study provides valuable information on the recurrence of TB and MDR-TB in Henan Province, China, although our study has limitations. We could not follow up all patients in the 30 counties that were in the baseline survey in 2001. Due to resource limitations, we could not evaluate DOTS in the Province. Some data were self-reported. Annual household income and socioeconomic status may have been underestimated.

We believe that our study has important implications for TB control in China and worldwide, especially for countries with high TB burdens [8, 9, 24]. High recurrence rates pose continuing threats to communities and challenge TB control programs. It is well known that MDR-TB has poor treatment outcomes [23]; these outcomes will be made worse by high recurrence rates. Poorer outcomes place a huge social, economic, and psychological burden on patients, families, and communities.

TB is a disease of poverty, and we think it should be a priority to alleviate the economic burden on TB patients. The Chinese government has designated MDR-TB as one of eight priority diseases eligible for 70% reimbursement in the country’s rural health insurance program. However, the reimbursement proportion may need to be increased, and additional financial assistance may need to be provided to ensure that the poorest and most vulnerable patients have access to care and are able to complete their treatment.

## Conclusion

This study demonstrated very poor long-term outcomes following treatment success for TB patients in a well-developed DOTS program (100% coverage in 2005) in China. From our findings, we conclude that in Henan, China, and potentially in other settings with similar drug resistance burdens [1–5, 7, 17, 25–28], the highest probability of TB recurrence occurs among the most vulnerable populations—patients with poor economic status and MDR-TB patients. Treatment outcomes reported in routine surveillance data with short follow-up time may overestimate treatment success. Treatment failure leads to morbidity and mortality and hampers TB control. Our findings also suggest that services and management for MDR-TB in China should be strengthened and NTP for MDR-TB patients should be scaled up urgently. Tailored interventions to control TB recurrence should be developed and evaluated in clinical trials.
